# Image perception of ice and snow tourism in China and the impact of the Winter Olympics

**DOI:** 10.1371/journal.pone.0287530

**Published:** 2023-06-23

**Authors:** Songying Liu, Quanen Guo

**Affiliations:** Department of Tourism Management, School of Tourism, Nanchang University, Nanchang, Jiangxi, China; Sichuan Agricultural University, CHINA

## Abstract

This study analyzes image perceptions of ice and snow tourism destinations in China. Using network text analysis on data from several online travel platforms such as Ctrip, Qunar, and Meituan, it further investigated how the Winter Olympics impacts destination image. Results reveal the following 1) The development patterns of ice and snow attractions in northern and southern China are different. 2) Ice and snow tourism destination imagination in China is generally positive. 3) The 2022 Winter Olympics has no significant influence on the image perception of ice and snow tourism destinations. The Winter Olympics increases tourists’ interest in ice and snow tourism, but the lagging development of service and management levels in scenic spots cannot match the sudden increase in visitors. This study provides a reference for researchers to recognize the image of China’s ice and snow tourism destinations and suggests ways for policymakers to promote such tourism attractions.

## 1. Introduction

Over the past 15 years, the process of travel decision-making has been transformed by the emergence of online user-generated content reviews [1]. Online reviews have gradually increased with the continuous development of big data in the Internet era. When deciding where to travel, travelers increasingly rely on online reviews [2]. As a result, the destination image, generated from online reviews, also affects tourists’ perceptions [3] and becomes the focus of scenic travel managers. Analyzing tourist destination image perception in response to online reviews is fundamental to scenic area planning and management.

The analysis of the image perception has received widespread attention in academia. Previous studies indicated the destination image was associated with tourists’ willingness to revisit [4, 5] and recommend [6], loyalty toward [7, 8], and satisfaction with the destination [3], as well as the social image construction of tourism destinations [9]. In recent years, although scholars have also examined the relevance of destination image to major sporting events [10], there are few studies focusing on destination image perception in ice and snow tourism.

Ice and snow tourism (IST) first appeared in Norway, Finland, Sweden, Russia, and other countries around 4,500 to 5,000 years ago [11]. IST combines sports, sightseeing, vacation, and business activities, which can generate economic benefits [12]. Meanwhile, IST, offering excitement and challenges, is popular worldwide. Compared with developed countries and regions, IST in China is emerging.

‘The China Ice and Snow Tourism Development Report (2022)’ states that the number of people participating in ice and snow recreation in China has increased from 170 million in 2017 to 254 million in 2021. IST in China has become increasingly popular in recent years. Stimulated by the Beijing Winter Olympics, more and more residents have increasing interests in ice and snow activities.

As the highest level of ice and snow sports event in the world, the Winter Olympic Games has a significant influence on constructing and spreading tourism destination image (TDI) [13–15]. The 2022 Beijing Winter Olympics is the first large-scale ice and snow event held in China. As the second economy in the world and a popular tourism destination, it is necessary to analyze the influence of the Winter Olympics on TDI in China.

Therefore, we used the ROST Content 6 software to crawl data from travelogues and reviews on popular websites such as Qunar, Ctrip, and Meituan. The data were used to analyze the image perceptions of China’s two main ice and snow tourism destinations from northern and southern regions. Specifically, we investigated the changes in image perceptions before and after the Winter Olympics. Finally, we offered suggestions to improve the core competitiveness of scenic spots and provided the theory reference of IST development.

This study contributes to the literature in two ways. 1) It considers the regional differences in China, comparing the influence of the Winter Olympics on TDI in northern and southern regions. 2) This study finds that the 2022 Winter Olympics did not significantly change tourists’ overall perceptions of destination image but generated more negative emotions.

The rest of this study is arranged as follows. Section 2 is the literature review. Section 3 is methodologies. Section 4 is research results and Section 5 is discussion, limitation, and future research.

## 2. Literature review

Scholars have conducted an in-depth analysis of the image of the destination [16]. It is obvious that the research of the destination image has been a relatively mature field. In the last decade, several review papers and meta-analyses were published on the topic of destination image research [17–19].

### 2.1 Tourism destination image

TDI consists of visitors’ trusts (Beliefs), opinions (Ideas), impressions (Impressions), and expectations (Expectations) [20]. TDI, influencing tourists’ behavior before, during, and after the trip [21–23], is a subjective perception of the destination by individuals [24–26]. It is widely acknowledged that the structure of TDI includes three layers: cognitive, affective, and idea [27]. Some studies found that both the overall image and affective image have influences on tourists’ behavioral intentions. Furthermore, affective factors have more influence on the overall image and future behavior of visitors [16, 28].

Scholars paid more attention to the impact of environmental changes on IST [29, 30] and the sustainable development strategies in the future [31–33]. Scholars analyzed the influence of accessibility [34] and industrial integration [35] on ice and snow tourism destinations. Most of them paid attention to the problems and development paths of IST [36], destination satisfaction [37, 38], and adaptability and utilization of regional ice and snow resources [39–41]. Although scholars have conducted further research on the economy and destination of IST in recent years, there are still few analyses on the image perception of ice and snow tourism destinations.

Destination image is widely regarded as a powerful management tool that can boost competitiveness in the tourism market [16]. Early literature considered the relationship between image and decision-making to be significant. This is because decision-makers act on their impressions of the situation rather than objective reality, resulting in potential visitors’ perceptions of tourism and recreation areas being influenced by this image [20]. This impression can significantly impact the development and ultimate success of a destination [42]. Regarding the stimulating factors, researchers believed that social media, destination tours, the image of the network platforms, that of the destination among social network members, online reviews, and the application of smart tourism both influenced the way tourists perceived the image of the destination [43–48]. A positive destination image enhances the credibility of the destination source, which affects tourists’ satisfaction with and attachment to the destination and their loyalty toward it [3, 7]. A negative or distorted evaluation of a destination increases tourists’ perceived risk, affects their willingness to revisit, and shapes the destination image, making it challenging to realize potential utilization or optimal economic development [42, 49]. Therefore, managers of tourist destinations attach great importance to the management and marketing of scenic spots. Managers seek to enhance the image of a destination and shape the cultural image of a country, region, or scenic spot through web design, market segmentation, and establishment of tourism service evaluation standards [50]. Additionally, online evaluation of destinations, destination movies, and other content can reduce the risk of unfavorable tourist decision-making [47, 48].

### 2.2 The impact of the Olympic Games

As a large-scale sporting event, the Olympic Games have significant impacts on regional economy, social culture [41, 51], social stability [52], and the art industry [53, 54]. Scholars have conducted extensive and in-depth research on the impact of the Olympic Games [55, 56].

The impact of sporting events on destination image has also received attention from scholars [57, 58]. It has concluded that sporting events have direct and indirect effects on the behavioral intentions of tourists [59–62]. However, regarding the impact of major sporting events on the image of destinations, the conclusion has not been reached the same yet [63]. Scholars with positive views found that sporting events can promote urban mobility, attract tourists, and improve the development of tourism [17]. Also, the practice of entertainment, aesthetics and escapism in the sporting event can enhance the image of the destination [64]. Scholars with a negative view found that misuse of public resources, increased consumption, destination marketing, and non-persistent historical issues seriously affect the image of the destination [65, 66]. The negative imagination generated by the Olympic Games is persistent and difficult to change and improve [16, 72].

**Fig 1:**
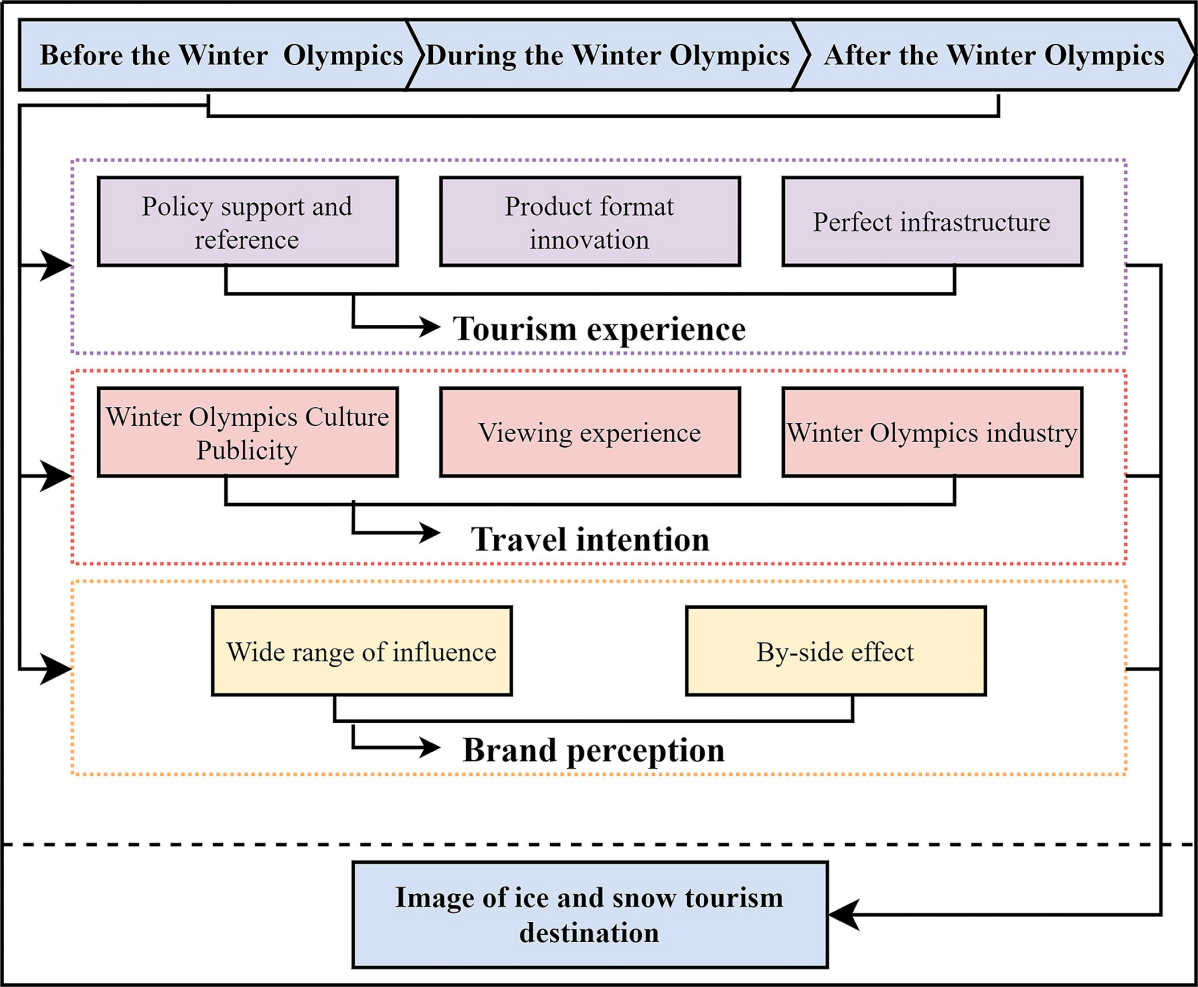
The impact path of the Winter Olympic Games on IST.

In summary, although there were many studies followed the impact of the Olympic Games on TDI, few studies focused on the influence of the Winter Olympics on TDI of ice and snow tourism destinations. As the largest international sports event, the Winter Olympic Games have the characteristics of large investment and wide influence, becoming the catalyst to improve the tourism image of the host country. Since the successful bid, the Winter Olympics have attracted the world’s attention and influenced the image of the destination before, during, and after the Winter Olympics [13, 67]. The impact of the Winter Olympics is not limited to the location of the event, it also has implications for other non-host regions [68]. The impact path of the Winter Olympics on ice and snow tourism destinations includes but is not limited to the following three points: 1) The related policy will promote the innovation of ice and snow products and the improvement of infrastructure, improving the tourist experience [69]. 2) The Winter Olympic Games, as a famous sports brand, has a wide range of radiation. Tourists’ perception of IST is sensitive to the by-side effect of the Winter Olympics [70, 71]. 3) The Winter Olympics culture [72], watching the experience of sports events [73], and the relative industry [74] can raise the Winter Olympics enthusiasm and form the image of the ice and snow tourism destination (Fig 1).

With the participation of 91 countries and 2,880 athletes, the 2022 Beijing Winter Olympics is the most watched Winter Olympics on digital media platforms and has been widely recognized by many international social media and experts, including NBC, IOC President Thomas Bach, and others. At the same time, there is also a wave of ice and snow in China. Before the 2022 Winter Olympics, 346 million people in China participated in ice and snow sports, which will effectively promote the development of ice and snow sports in the world. It can reflect the authority and influence of large sports events and is persuasive to select them as representative cases.

## 3. Methodologies

### 3.1 Study area

This study takes Yabuli (Heilongjiang) and Xiling (Sichuan) attractions as cases. These attractions are famous and located in northern and southern China, respectively. Moreover, there are more tourists in Yabuli and Xiling than in others.

The research object is divided into two subjects. The first is Yabuli ice and snow tourism destination, which includes the Yabuli New Sports Commission Ski Resort, Yabuli Ski Resort, Yabuli Qingyun Ski Resort, Yabuli Yawangs Ski Resort (Yabuli International Convention and Exhibition Center), Yabuli Sunshine Resort, Yabuli National Forest Park, and New Yabuli Ski Resort. The second is the Xiling ice and snow tourism destination, including the Xiling Snow Mountain, Xiling Snow Mountain Ski Resort, Xiling Dafei Water Scenic Area, and Xiling National Forest.

### 3.2 Research method

Content analysis is an important method of textual research [75]. It transforms non-systematic and non-quantitative symbolic content (such as text, images, pictures, etc.) into data and uses it for quantitatively analyzing the content of the material. Content analysis contains high-frequency word analysis, social-semantic network analysis, and sentiment analysis. In the past, scholars have mainly used questionnaires [76], LDA data analysis [77, 78] and descriptive statistics methods to investigate destination image perception. However, these methods have weaknesses such as low data collection, inflexibility, and narrow focus. We adopt a combination of qualitative and quantitative methods to investigate the TDI of IST in China. Referring to Popping [79], Diesner [80], and Wong’s [81] research on network text analysis, and Liu’s [82] on high-frequency word analysis, we used ROST Content Mining 5.8 text analysis software to collect relevant web travelogue texts and word processing of plain text web travelogues to examine travelers’ emotional image and overall perceived TDI.

**Fig 2:**
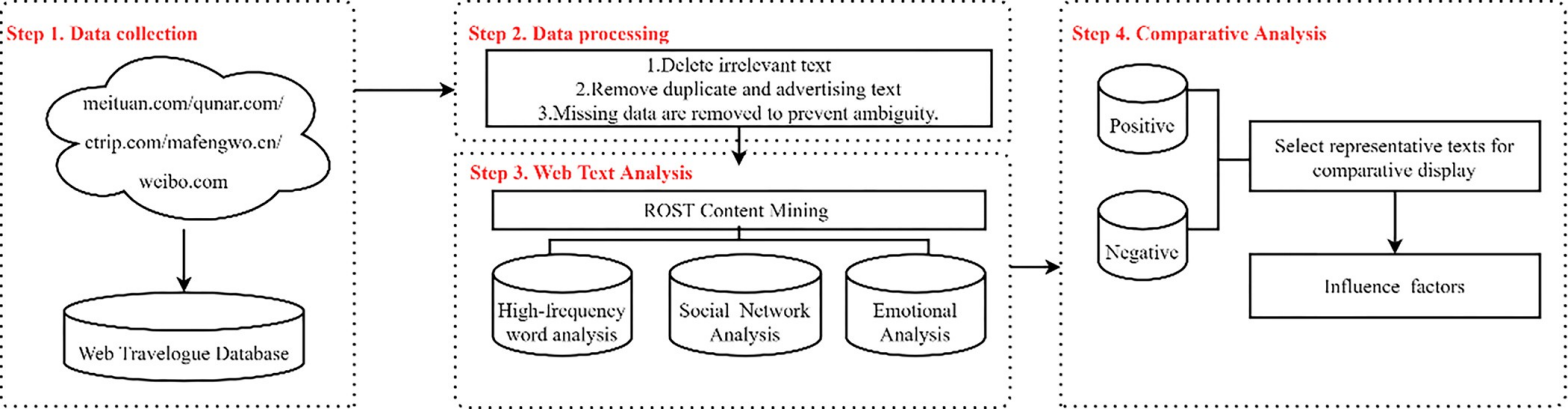
Research flow.

Fig 2 is the research flow in this study. First, this study applies an octopus data crawler, with Qunar, Ctrip, Meituan, and mafengwo as the main data resources, and takes the comments of Weibo as a reference. The search keywords are the destination names which are listed in 3.1. Due to the large data set, this study used the text analysis tool named ROST Content Mining 5.8. To make the results more accurate, we revised and cleaned data. The details of criterion were shown in 3.3. Thirdly, high-frequency word analysis, social-semantic network analysis, and sentiment analysis were used to reveal the time trend of image perception changes in ice and snow tourism destinations. Finally, the reasons for changes in the image perception of ice and snow tourism destinations were disclosed.

### 3.3 Data resources

Considering the repetition of text and the reduction of text processing accuracy by ad-hoc comments, this paper used Octopus to process repetitive comments and manually cleaned the remaining comments, while considering the timeliness of visitor comments and data processing needs. Specifically:

1. Delete irrelevant text (Text that is not relevant to the topic and text that has no real meaning).
2. Remove duplicate and advertising text.
3. Missing data are removed to prevent ambiguity.

The data sources for the image analysis of destinations before the Winter Olympics referred to previous data sources [83], this study chose Ctrip, Meituan, Qunar, mafengwo, and Weibo. Octopus software was used to obtain tourists’ evaluations of Yabuli and Xiling as ice and snow tourism destinations. Because the literature on such tourism has increased significantly since 2018, the reviews considered in this study are from January 2018 to December 2021. A total of 8,968 comments were processed and counted from five platforms, 3,910 for Yabuli and 5,058 for Xiling.

The data sources of destination image analysis after the Winter Olympics from February to April 2022 were selected for comparative analysis. By selecting the main text and deleting that concerning propaganda advertisements, a total of 673 tourist reviews of seven ice and snow tourism destinations were screened out from February to April 2022, including 475 for Xiling, Xiling Ski Resort, 198 for New Yabuli Ski Resort, Yabuli Sunshine Resort, Yabuli Ski Resort, and Yabuli New Sports Committee.

## 4. Results

### 4.1 Destination image before the Winter Olympics

#### 4.1.1 Comparative analysis of high-frequency words in different destinations

A comparative analysis of the top 30 words for the two places reveals that Yabuli and Xiling provide tourists with a similar image as tourist destinations (Table 1). Tourists’ perception of these as tourism destinations were divided into four categories: distinguishing factors (snow scenery, skiing, cloud sea), feeling factors (convenient, happy, beautiful), cost factors (entrance fee, cost-effectiveness), and other factors (queuing, coaching). The scenic features of the two places were considered highly similar. Both are ice and snow tourism destinations known for winter skiing. Nevertheless, there are differences in Yabuli’s scenic features including hot springs and resorts. By contrast, Xiling’s scenic features include natural landscapes such as the Sea of Clouds, Sun Moon Lake, and the Yin Yang boundary. The development paths of these two places also differ.

**Table 1:** Comparative analysis of high-frequency words for Yabuli and Xiling.

Yabuli New Sports Commission Ski Resort				Xiling Snow Mountain			
Serial number	Glossary	Wordiness	Word Frequency	Serial number	Glossary	Wordiness	Word Frequency
1	Skiing	1408	verbs	1	Snowy Mountains	825	noun
2	Coaches	1006	noun	2	Ropeways	824	noun
3	Ski Resorts	985	noun	3	Scenery	664	noun
4	Yabuli	785	noun	4	Skiing	520	verbs
5	Services	512	verbs	5	Landscapes	518	noun
6	Fun	421	verbs	6	Scenic areas	501	noun
7	Experience	366	noun	7	Fun	489	verbs
8	Happy	353	verbs	8	Queuing	401	verbs
9	Harbin	350	noun	9	Sun and Moon	389	noun
10	Snow Trails	347	noun	10	Chengdu	357	noun
11	Hours	346	noun	11	Hours	345	noun
12	Tipping	320	noun	12	Weather	336	noun
13	Playful	291	verbs	13	Hilltop	336	noun
14	Scenery	268	noun	14	Projects	325	noun
15	Value for money	223	noun	15	Tickets	319	noun
16	Overall	222	noun	16	Gondola	305	noun
17	Time	218	noun	17	Experience	303	verbs
18	Interesting	209	adjectives	18	Worthwhile	296	verbs
19	Junior	204	distinguishing words	19	Time	288	noun
20	First time	201	numerals	20	On the Mountain	283	noun
21	Hot Springs	196	noun	21	Skiing	274	noun
22	Sports Commission	184	noun	22	Convenient	269	adjectives
23	Local	179	noun	23	Sea of Clouds	267	noun
24	Personnel	174	noun	24	Up the hill	265	verbs
25	Convenient	174	adjectives	25	Happy	240	verbs
26	Tourism	168	verbs	26	Beautiful	237	adjectives
27	Facilities	160	noun	27	Interesting	237	adjectives
28	Slipway	159	noun	28	Overall	231	noun
29	Recommendations	159	verbs	29	Local	230	noun
30	Attitude	155	noun	30	Recommendations	221	verbs

Regarding the perception factors, the two places have similarities, and tourists have commented positively using the words ‘happy’ and ‘worth it’. These words suggest that the overall tourism destination image is relatively good and that the novelty of ice and snow tourism destinations appeals to tourists. Regarding cost factors, tipping in Yabuli is mentioned several times, meaning that tourists have doubts about local tipping. The frequency of ‘entrance fees’ is high in the reviews of Xiling. For other factors, ‘queuing’ and ‘hours’ appear more often for Xiling, indicating that tourists attach importance to the time spent on the tour, and the queuing time in the scenic spot impacts tourists’ psychological and subjective evaluation. For Yabuli, the word ‘coach’ ranked second (1,006 times), meaning that tourists highly depended on the coach to develop play activities. From the analysis, we can summarize that the critical factor that attracts tourists is the distinguishing feature of the scenic spot, which is its unique cognitive image. The unique experience offered by the two scenic spots is an essential feature that attracts tourists. Visitors also pay close attention to costs, activities, and other factors.

#### 4.1.2 Comparative analysis of social-semantic networks in different destinations

High-frequency words can reveal the main aspects and contents of tourists’ concerns, but the internal logic and hierarchy of the text cannot be studied in detail. By constructing a co-linear matrix of high-frequency words, we can extract critical information, perform visual analysis, and obtain the semantic structure of these two places to reveal the image perception of these tourist destinations. Specifically, the blue square indicates the high-frequency words node, and the line between the nodes indicates the shortest distance between them in the matrix sense, the smaller the distance, the smoother the message communication between them.

**Fig 3:**
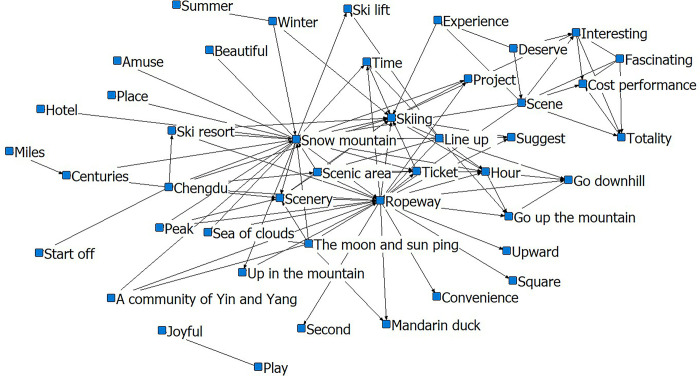
Social-semantic network analysis diagram for Xiling. Notes: The top 3 most connected nodes: Snow mountain, Skiing, Ropeway.

In Fig 3, skiing and ropeway are taken as the core, with skiing-snow mountain, skiing-ropeway, skiing-scenery, and skiing-uphill as the relational chains closely linked to the body. The ‘ropeway-scenery-snow mountain-skiing-time queue going up the mountain-skiing area’ network forms an interlocking relationship network, indicating a corresponding logical relationship between these words and the social activities formed by multiple words, and they are closely connected. The ‘scenery-fun-interesting-worthy-experience’ relationship network demonstrates that scenic features influence the creation of tourists’ subjective perceptions. The characteristics of the outbound tourist groups are drawn from the ‘kid-happy-play’ relationship chain.

**Fig 4:**
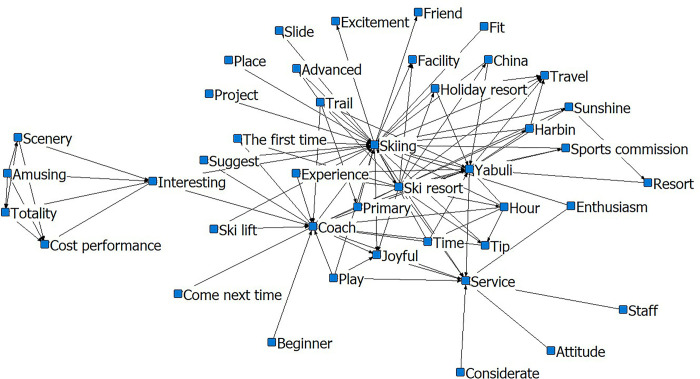
Social-semantic analysis diagram for Yabuli. Notes: The top 3 most connected nodes: Skiing, Yabuli, Ski resort.

In Fig 4, a core circle forms with ‘skiing-Yabuli-services’ and other semantic words revolving around it. The terms ‘happy’, ‘instructor’, and ‘ski resort’ are close to the sub-core circle, and the relationship chain supplements the logic and content of the core circle. Words such as ‘tip’, ‘advice’, ‘facilities’, ‘attitude’, and ‘resort’ serve as peripheral circles to complete the chain of relationships between the first two layers. The relationship network formed by ‘overall-value-for-money-interesting-scenery’ reflects the tourists’ overall perception of the Yabuli ice and snow tourism destination.

#### 4.1.3 Comparative analysis of sentiment analysis in a different destination

Tourism sentiment analysis (SA) is an intuitive way to obtain tourists’ perceptions of the image of a tourist destination. A negative emotional experience may damage the image of the destination in tourists’ minds, thus affecting their willingness to revisit. A positive affective image means that tourists are more satisfied with local services, infrastructure facilities, the environment, and other factors in the destination, which leads to tourists’ positive emotions.

This study used the ROST EA SA method to analyze and compare the sentiments expressed in tourists’ comments on the two scenic spots (Table 2). The proportion of positive sentiments for both ice and snow tourism destinations is higher than the negative sentiments, indicating that tourists have a more positive attitude toward these scenic spots. Such sentiments represent that tourists have a favorable perception of the two destinations. However, some expressed negative emotions, and the underlying reasons may be the key factors hindering tourists from traveling to these destinations. The keywords extracted from the reviews expressing negative emotions reveal problems such as the poor attitude of scenic area personnel, poor environment, and old ski equipment being used in Xiling. For Yabuli, the problems were disorderly management, indiscriminate tipping, and poor customer service experience.

**Table 2:** Comparison of the emotional image before the Winter Olympics.

Name	Positive sentiment	Neutral sentiment	Negative sentiment	Total number of statements
Yabuli ice and snow tourism destination	63.27%	0.82%	35.91%	3907
Chengdu Xiling ice and snow tourism destination	61.91%	0.97%	37.12%	5057

Meanwhile, other reviews evaluated the same problems differently. Take the Yabuli tipping problem as an example. Some tourists claimed that the coach tips were high and there were problems of poor service.

*“I have no talent for skiing and skating. I spent 200 RMB to hire a coach and then went back the next day and still did not know anything. As for the venue, I am too much of a layman to comment, but the site staff is truly greedy. We intended to take the cableway down and were reprimanded by the staff for being too slow. The unlimited service money is the priority.”*

However, there is an explanation for the tipping issue:

*‘Finally, to say a few words about the coach’s tips, many posts state that snow coaches ask for tips. My coach also asked me for tips, and I did give him one. Giving tips or not is voluntary, not mandatory. The reason that I did it is I learned from the relevant people that the coach only gets 30% of the coaching fee.’*

*‘It should be pointed out that the coach here is one-to-one, only responsible for the safety of one student, not other pedestrians; 3 hours 240 yuan, the cost is more expensive than the ticket. Why should we have a coach? First, for safety, we have no skiing experience. Skiing is a sport with certain dangers. Go out and play safely first. Second, without a coach, you can’t go to the intermediate track. The beginner track is like a bathhouse; you can’t move at all and the whole point of skiing is lost. My coach also proved to be very good. I also learned a lot. In just under three hours, I learned to master the plow brake, plow turn, and parallel turn. I’m still satisfied with my learning progress, although I fell twice in the middle; the coach was scared the students were injured, because [if they are] the coach will be fined a lot of money.’*

Many tourists complained about the charge. However, some tourists’ attitudes toward tipping changed from negative to positive. Thus, further analysis of the comments on the issue of tipping in Yabuli can provide a more comprehensive image of the tourist destination.

Regarding negative comments, the most frequently mentioned complaint for both places concerned safety, particularly regarding ski-induced falls.

*‘The overall snow slope is perfect. After all, the novice, all that is called a tragedy!’*

*‘I fell at the beginning in front of the ski resort, the security came to help me and said they are specialized in helping people… However, when I fell again, the security would not help… and then I slid too fast on the slope and fell heavily on the ground and crawled up, struggling for ten minutes, no one came to help me, maybe falling is more common. To get up, hands and skin are broken, so bring gloves!’*

At present, there are no specific yearly statistics regarding how many people are killed or injured while skiing in China. As a high-risk sport, scenic spots should improve the relevant infrastructure, equip the facilities with appropriate medical resources, install monitoring equipment, and assign security personnel to maximize the safety of tourists. Whether in Yabuli or Xiling, ice, and snow tourism destinations should pay attention to the safety of tourists.

### 4.2 Destination image after the Winter Olympics

Ice and snow sports have been considered as the ‘flower of the high mountains’, but the number of participants remains low. Since the opening of the Winter Olympics in Beijing, China, the Baidu index of IST has soared, doubling from 110 in 2021 to 230 in 2022. The Beijing Winter Olympics bid has extensively promoted the development of winter sports and inspired more than 300 million people to participate. As the most representative sports event, the Winter Olympics is of great social significance to investigating the impact of sports events on ice and snow tourism destinations.

#### 4.2.1 Analysis of high-frequency words

Text analysis using ROST CM established the characteristic word frequencies of traveler reviews for Xiling and Yabuli after the 2022 Winter Olympics. The top 30 high-frequency words for each destination were obtained to compile a word frequency table, which was compared and analyzed against the characteristic high-frequency words from January 2018 to December 2021 (Table 3). Generally, a higher frequency of a feature word indicates that tourists perceive the feature more obviously. The high-frequency feature words also tend to capture tourists’ perception of the destinations.

**Table 3:** High-frequency words for Xiling before and after the Winter Olympics.

List of high-frequency words for snow and ice tourism destinations for Xiling						
Serial number	Ahead of the 2022 Winter Olympics			After the 2022 Winter Olympic Games		
	Characteristic words	Proportion	Wordiness	Characteristic words	Proportion	Wordiness
1	Snowy Mountains	0.1630	noun	Snowy Mountains	0.1621	noun
2	Ropeways	0.1629	noun	Skiing	0.1578	verbs
3	Scenery	0.1312	noun	Queuing	0.1536	verbs
4	Skiing	0.1028	verbs	Scenic areas	0.1284	noun
5	Landscapes	0.1024	noun	Chengdu	0.1052	noun
6	Scenic areas	0.0990	noun	Ropeways	0.0968	noun
7	Fun	0.0966	adjectives	Landscapes	0.0905	noun
8	Queuing	0.0792	verbs	Hours	0.0757	noun
9	Sun and Moon	0.0769	noun	Downhill	0.0631	verbs
10	Chengdu	0.0705	noun	Skiing	0.0055	noun
11	Hours	0.0682	noun	Up the hill	0.0589	verbs
12	Weather	0.0664	noun	Experience	0.0589	noun
13	Hilltop	0.0664	noun	Time	0.0547	noun
14	Projects	0.0642	noun	Services	0.0505	verbs
15	Tickets	0.0630	noun	Beautiful	0.0505	adjectives
16	Gondola	0.0603	noun	Tickets	0.0505	noun
17	Experience	0.0599	verbs	Child	0.0505	noun
18	worthwhile	0.0585	verbs	Fun	0.0484	adjectives
19	Time	0.0569	noun	Convenient	0.0484	adjectives
20	On the Mountain	0.0559	noun	Gondola	0.0484	noun
21	Skiing	0.0541	noun	Projects	0.0442	noun
22	Convenient	0.0531	adjectives	Weather	0.0442	noun
23	Sea of Clouds	0.0527	noun	Facilities	0.0442	noun
24	Up the hill	0.0523	verbs	Hilltop	0.0442	noun
25	Happy	0.0474	adjectives	Sea of Clouds	0.0421	noun
26	Beautiful	0.0468	adjectives	Sun and Moon	0.0421	noun
27	Interesting	0.0468	Adjectives	Scenery	0.0421	noun
28	Overall	0.0456	noun	Go up	0.0400	verbs
29	Local	0.0454	noun	Snowscape	0.0378	noun
30	Recommendations	0.0436	verbs	Playful	0.0357	verbs

Note: Frequency = word frequency / total number of words in the sample.

Comparing the high-frequency words before and after the Winter Olympics, the occurrence proportion of ‘snow mountain’ and ‘skiing’ did not change significantly, indicating tourists’ perception of the basic attributes in Xiling remained the same. The occurrence proportion of the word ‘queue’ increased from 0.0792 to 0.1536, indicating the number of tourists visiting Xiling changed significantly after the Winter Olympics, and tourists’ concern about the queues increased. While the counts of adjectives such as ‘beautiful’, ‘convenient’, and ‘fun’ decreased compared with the previous ranking, the occurrence proportion of these three words was similar. It indicates that tourists’ image of Xiling remained the same after the Winter Olympics. The words ‘cable car’, ‘ski resort’, and ‘cable car’ remained at the top of the list, indicating that tourists continued to pay much more attention to the public infrastructure of tourist destinations regardless of the impact of the Winter Olympics.

Comparing the high-frequency words for Yabuli as an ice and snow tourism destination before and after the Winter Olympics, we found that the frequency ranking of ‘Yabuli’, ‘skiing’, ‘ski resort’, and ‘ice and snow’ did not change significantly (Table 4). The results indicated that tourists still consider the characteristic words mentioned above. The frequency of ‘coach’ decreased significantly after the Winter Olympics, indicating that tourists were less concerned about it. The related word ‘tipping’ did not appear after the Winter Olympics, suggesting that the tipping problem has been rectified. The occurrence frequency of ‘Yabuli’, ‘sports committee’ (new sports committee ski resort), ‘Sunshine’ (Yabuli Sunshine Resort), and ‘resort’ ranked high, indicating that tourists were increasingly attracted to the scenic features after the Winter Olympics.

**Table 4:** High-frequency words for Yabuli before and after the Winter Olympics.

High-frequency words list for Yabuli IST tourism destination						
Serial number	Ahead of the 2022 Winter Olympic Games			After the 2022 Winter Olympic Games		
	Characteristic words	Proportion	Wordiness	Characteristic words	Proportion	Wordiness
1	Skiing	0.3601	verbs	Yabuli	1.7272	noun
2	Coaches	0.2572	noun	Skiing	1.6262	noun
3	Skiing	0.2519	noun	Skiing	1.5757	verbs
4	Yabuli	0.2007	noun	Harbin	0.6868	noun
5	Services	0.1309	verbs	Sports Commission	0.5151	noun
6	Fun	0.1076	verbs	Tourism	0.4797	noun
7	Experience	0.0936	noun	Resort area	0.3989	noun
8	Happy	0.0902	verbs	Snow Trails	0.3787	noun
9	Harbin	0.0895	noun	Snow and ice	0.3232	noun
10	Snow Trails	0.0887	noun	China	0.3181	noun
11	Hours	0.0884	noun	Sunshine	0.3030	noun
12	Tipping	0.0818	noun	Holiday Village	0.2373	noun
13	Playful	0.0744	verbs	Hotels	0.2020	noun
14	Scenery	0.0685	noun	Scenic areas	0.1868	noun
15	Value for money	0.0570	noun	Hilltop	0.1818	noun
16	Overall	0.0567	noun	Architecture	0.1818	noun
17	Time	0.0557	noun	Hours	0.1767	noun
18	Interesting	0.0534	adjectives	Coaches	0.1616	noun
19	Junior	0.0521	distinguishing words	Heilongjiang	0.1616	noun
20	First time	0.0514	numerals	Kilometers	0.1565	noun
21	Hot Springs	0.0501	noun	Experience	0.1515	noun
22	Sports Commission	0.0470	noun	Arrival	0.1414	noun
23	Local	0.0457	noun	Junior	0.1414	verbs
24	Personnel	0.0445	noun	Snow	0.1414	noun
25	Convenient	0.0445	adjectives	Web	0.1313	noun
26	Tourism	0.0429	verbs	North East	0.1262	noun
27	Facilities	0.0409	noun	Tickets	0.1262	noun
28	Slipway	0.0406	noun	Hot Springs	0.1212	noun
29	Recommendations	0.0406	verbs	Suitable for	0.1212	adjectives
30	Attitude	0.0396	noun	Winter	0.1161	noun

Note(s): Frequency = word frequency / total number of words in the sample.

#### 4.2.2 Social-semantic network analysis

**Fig 5:**
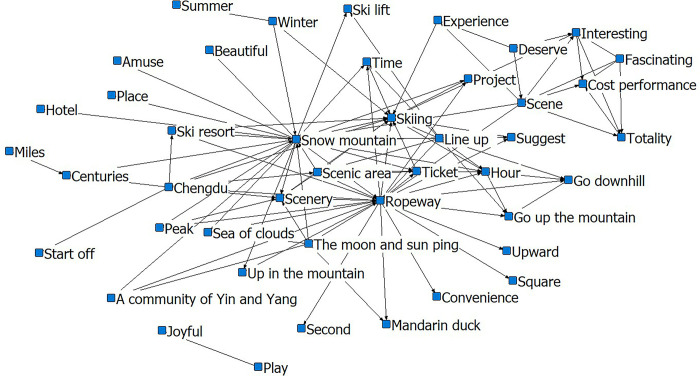
Social-semantic network analysis for Xiling pre-Winter Olympics. Notes: The top 3 most connected nodes: Snow mountain, Skiing, and Ropeway.

**Fig 6:**
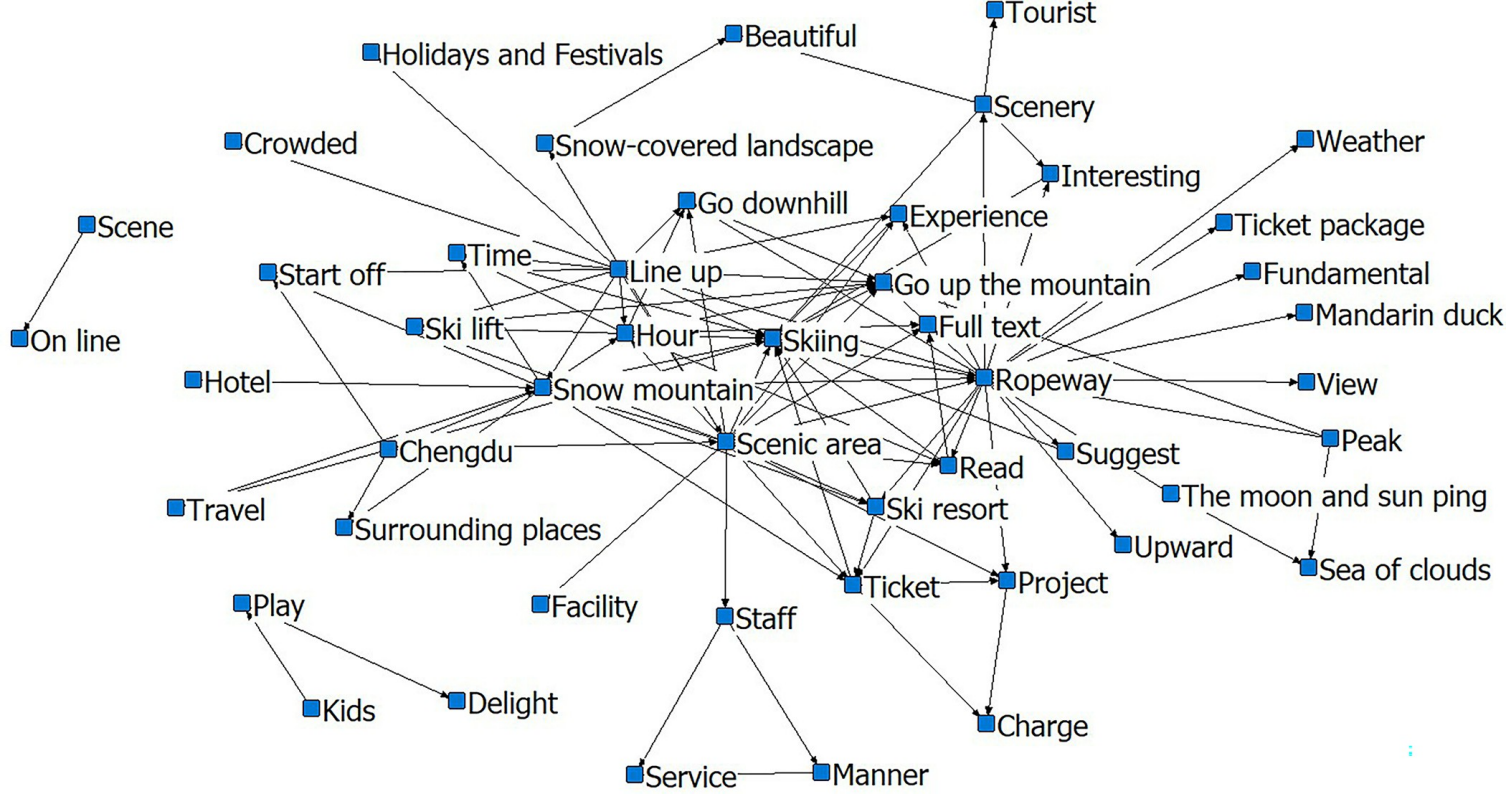
Social-semantic network analysis for Xiling post-Winter Olympics. Notes: The top 3 most connected nodes: Skiing, Ropeway, and Go up the mountain.

Although the core of the social-semantic network analysis chart is ‘skiing’, the network changed after the Winter Olympics (Figs 5 and 6). Instead of the ‘cost-fun-worthy-experience-fun’ network, the outer layer comprises ‘beautiful’, ‘fun’, and ‘happy’. The relationship network after the Winter Olympics is divided into three types. The first is the ‘cloud-sea-sunrise-surrounding-project-ski-resort-snow mountain’ relationship network. From this layer, we can conclude that tourists pay significant attention to the landscape characteristics of Xiling Snow Mountain. The ‘Sea of Clouds’, ‘sunrise’, and ‘skiing’ are representative of the combination of sceneries that embody the uniqueness of Xiling as one of southern China’s ice and snow attractions. The second is the ‘scenery-beautiful-snow-scenery-time’ relationship network, mainly representing tourists and their subjective feelings during the visit. The third is the ‘scenery-ropeway-go-up-mountain-hour-ticket’ relationship network. This network is closer to the core layer than the queuing network before the Winter Olympics. The connection density is higher, which suggests an increase in the number of visitors to Xiling after the Winter Olympics. Visitors were also concerned about the time spent going up and down the mountain. Moreover, the semantic network analysis reflected problems such as crowding and the difficulty of queuing.

**Fig 7:**
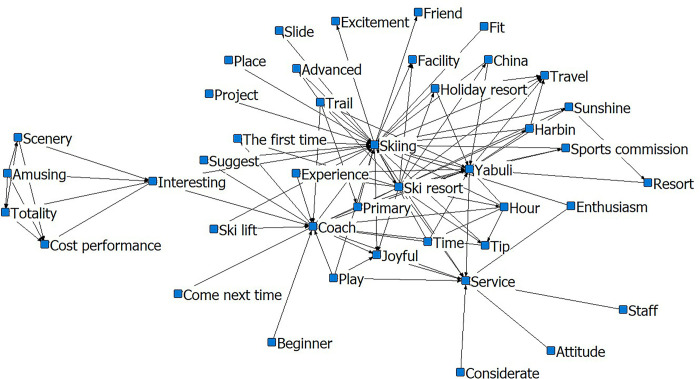
Social-semantic network analysis of Yabuli pre-Winter Olympics. Notes: The top 3 most connected nodes: Skiing, Yabuli, and Ski resort.

**Fig 8:**
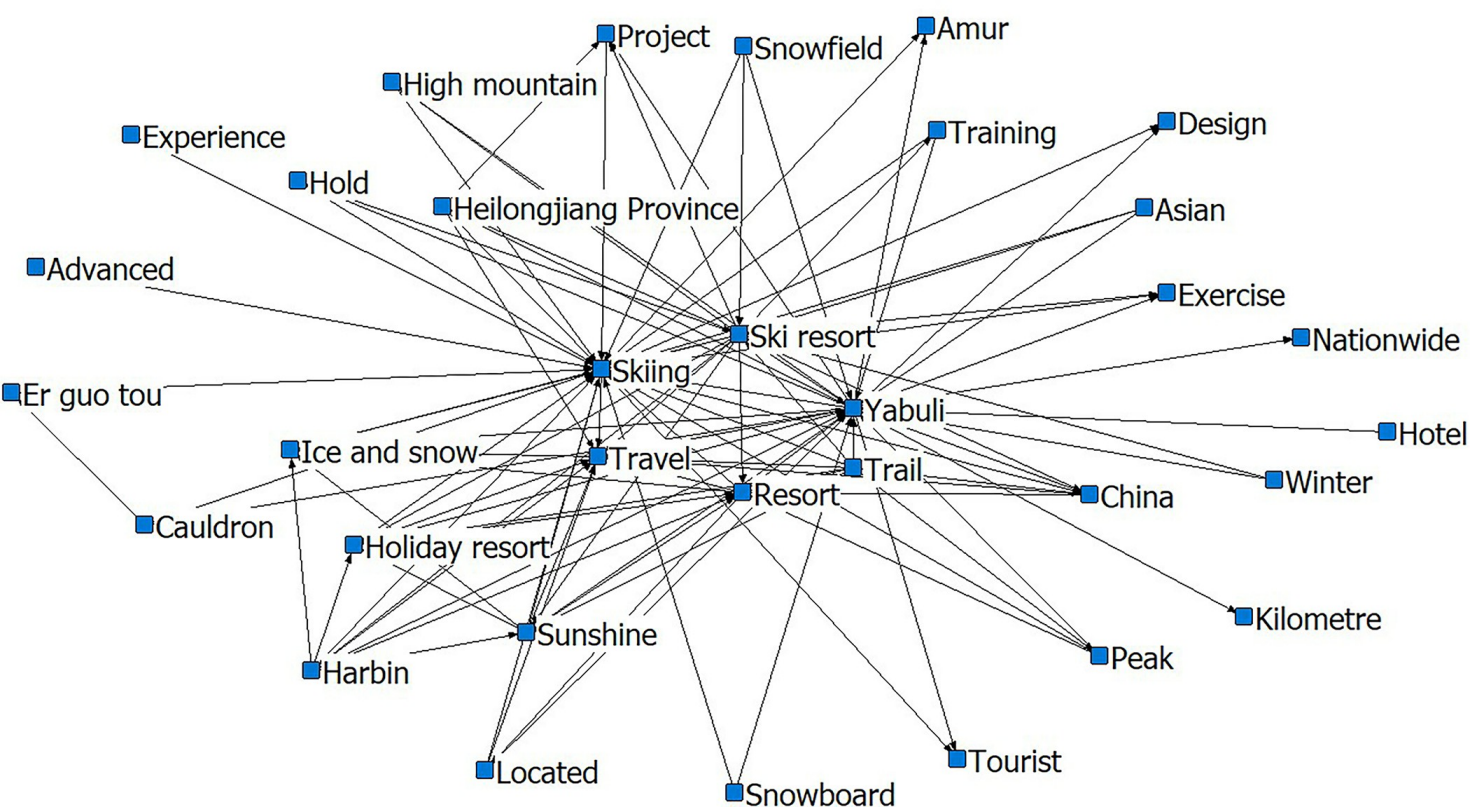
Social-semantic network analysis for Yabuli post-Winter Olympics. Notes: The top 3 most connected nodes: Skiing, Yabuli, and Travel.

After the Winter Olympics, the social-semantic network diagram of the Yabuli ice and snow tourism destination formed three circles. The inner core consists of ‘skiing’, ‘Yabuli’, and ‘a resort’. The sub-core is ‘sunshine’ ‘snow tracking’, ‘tourism’, ‘sports committee’ ‘Harbin’, and ‘ski resort’. The sub-core is primarily the name of the scenic spots, indicating that tourists are deeply concerned about the perception of and attention to these. Remarkably, the word ‘coach’, which is in the core circle before the Winter Olympics, moves to the peripheral circle thereafter. Moreover, the word ‘tip’ does not appear in the post-winter Olympics social-semantic network diagram, indicating that the problem of coaching and tipping in Yabuli has improved, been accepted, and recognized by most tourists (Figs 7 and 8).

#### 4.2.3 Sentiment analysis after the Winter Olympics

SA is an important research direction in natural language processing. It aims at mining the emotional viewpoint to be expressed by the text and classifying it by emotional tendency. We used ROST EA to analyze the sentiment of the post-Winter Olympics texts and extract representative texts [38].

Before the Winter Olympics, the tourists’ positive sentiments percentage of the whole reviews in Yabuli is 63.27%, while in Xiling is 61.91% (Tables 5 and 6). These positive comments use the adjectives ‘happy’, ‘fun’, and ‘interesting’ to describe Yabuli and Xiling scenic spots. Furthermore, the expected negative comments include common phrases such as ‘expensive consumption’, ‘poor infrastructure’, and ‘bad service attitude’. However, it should be noted that tourism attitudes are influenced by tourists’ demand for tourism consumption, related experiences, and personal perceptions. There are subjective differences in the evaluation of the same aspect.

**Table 5:** Comparison of the emotional image after the Winter Olympics.

Name	Time	Positive sentiment	Neutral sentiment	Negative sentiment	Total number of statements
Yabuli ice and snow tourism Destination	Before the Winter Olympics	63.27%	0.82%	35.91%	3907
	After the Winter Olympics	57.58%	1.52%	40.90%	198
Xiling ice and snow tourism Destination	Before the Winter Olympics	61.91%	0.97%	37.12%	5057
	After the Winter Olympics	49.26%	1.26%	49.48%	475

**Table 6:** Representative positive and negative evaluation texts.

	Positive Evaluation	Negative Evaluation
Yabuli ice and snow tourism Destination	It’s worth going, the snow park lift facilities are good quality, the price is not too expensive, and the intermediate snow track is about 1 km long, ski down more comfortably.	Today’s theme is skiing at the new Sports Commission ski resort in Yabuli. They have hired a private instructor for each of the kids for 240 two hours each. The experience was bad. In the beginning, they did not let the two warm up and did not teach them to turn the brakes on the beginner track. As soon as they came up, they took the children directly to the gondola on the intermediate road and came down after a lap slide experience. The child said it was too cold to play. It was only half an hour before and after, and then they asked for tips, which disgusted me that the ski ticket was 280 for on-site purchase, 138 for Ctrip, and 120 for local people.
	The weather in the Northeast is so cool, it’s like a refrigerator. It feels super good to cross the New Year here, praying for 2021 to come true. Started my first ski trip to Yabuli in the New Year, very happy day, and all the services are very satisfactory.	This time to Yabuli skiing, with the group to go, travel agency pit, and a bunch of self-funded projects. What we went to was not the new sports committee written in the contract, but sunshine ski resort. The primary ski slopes open to tourists are not as big as Liaoyang Gongchangling ski resorts. The temperature here is shallow, hands cannot take out, and a group of Anhui friends is in the hall until the return journey.
Xiling ice and snow tourism Destination	It is really worth going to play ah, Sichuan locals and next door to Chongqing friends feel very nice! Sitting on the ropeway, the scenery is stunning, the world in front of you in silver, as if in a fairy tale world. The first section of the ropeway to the Yuanyang pool, more people, play a lot of places, and the visitor center in the snow is simply beautiful. You can also take a lot of good pictures!	Service is not good. No one answered the customer service number if you had a question. There was no one to keep order in the queue at the attraction. The environment is ok, but the service is terrible.
	Xiling Snow Mountain is worth a visit! The snow is beautiful! It’s fun! Buy a set of tickets for parents and children! A family to play to buy a set of tickets is cost-effective, with children not tired. Scenic queuing orderly! Buy tickets online, do not have to queue on site, very time saving, to the site cell phone scan code to apply for the mountain queue number, or pay attention to the Xiling Snow Mountain public number in advance, and apply directly to the mountain queue number, very convenient.	One person just went less than a week. Just drop the price to 188, but also send two hours of skiing, the price difference is so big, garbage. The feeling of being cheated, we five people went to a loss of 600, garbage. On the ropeway, the scenery can still be. There is nothing else to play. Skiing is also dead expensive, and things are all rented separately. I have not seen the snow and can play under it, but be sure not to buy expensive ones!

After the Winter Olympics, tourists’ positive sentiments displayed a downward trend in both destinations. The number of positive sentiments for Xiling is less than half, at 49.26%. It indicates that tourists’ satisfaction of overall perceived has decayed, even though the Winter Olympics boosted the development of IST. This interesting phenomenon results from the difficult reservation and expensive tips in Yabuli, while the high price of tickets, chaotic order, and poor service in Xiling.

However, no matter before or after the Winter Olympics, the number of positive sentiments is more than the negative sentiments in Yabuli. Most tourists expressed that they felt more satisfied with Yabuli. Previously, the most outstanding problem in Yabuli was the tipping issue. Before the Winter Olympics, the negative sentiment heavily emphasized the issue of tipping and unreasonable fees. After the Winter Olympics, the number of such comments decreased. The positive sentiment rate regarding Xiling ice and snow tourism destination decreased significantly after the Winter Olympics. Most negative sentiments were caused by expensive ticket prices, queues, disorder, and poor service. In particular, the queuing problem is mentioned many times in the negative comments. This indicates that tourists attach great importance to this issue and complain about the crowd management of the scenic spots.

## 5. Conclusions and discussion

### 5.1 Conclusions

This study analyses the online text of the two representative ice and snow tourism destinations, Yabuli and Xiling. The impact of the 2022 Winter Olympics on these two destinations was compared and the following conclusions were drawn.

First, the analysis of high-frequency words reveals that the development pattern and the sceneries in these two destinations differ. Xiling, in southern China, has a classic landscape. Tourists visit Xiling for skiing and vacation during winter, while they come for rafting, climbing, watching the sunrise, and enjoying the Sea of Clouds during other seasons. Meanwhile, as the representative ski resort in Northern China, Yabuli provides ice sports and high-quality snow for a longer duration. In other seasons, the attraction can develop as a leisure place based on hot springs and forest resources.

Second, tourists’ image perceptions of these ice and snow attractions are more positive than negative. In other words, tourists are generally satisfied with Xiling and Yabuli as these types of destinations. However, negative emotions must be addressed. The expensive tickets, poor service, and disorderly queues are problems experienced by tourists in both destinations. Tourists are concerned about the queuing, infrastructure, and tipping problems, particularly for Yabuli. The analysis of emotions reveals that many tourists’ comments have shifted from positive to negative or vice versa, demonstrating that such sentiments are the product of subjective feeling, which is malleable.

Third, sporting events do not significantly influence the image perception of ice and snow tourism destinations. The hosting of the Winter Olympics is closely related to the image perception of such destinations. Although tourists’ interest enhances, positive impacts from the Winter Olympics are reduced by counteraction from the lagged and underdeveloped infrastructure. These attractions’ poor service cannot meet tourists’ demands. Moreover, many visitors express negative emotions, which have adverse effects on tourists’ decision-making and the sustainable development of these scenic spots.

### 5.2 Implications

#### 5.2.1 Theoretical implication

This study is an early research focusing on the image changes of ice and snow tourism destinations before and after the Winter Olympics. Different from other research [63], this study finds that hosting the Winter Olympics has a significant positive effect on public interest in IST, but the image perception of the destination can also influence by factors such as the infrastructure, management services, attitudes, and consumer prices.

Furthermore, this study investigates the relationship between TDI of ice-snow tourism attraction and the Winter Olympics in non-hosting cities. After the 2022 Winter Olympics, China put forward slogans such as ‘300 million people participate in ice and snow sports’, ‘ice and snow are also golden mountains and silver mountains’. However, this study finds that the current development of IST in China is not optimistic from the perspective of tourist experience. Negative factors such as high prices, poor service, and imperfect infrastructure will increase the travel expense and time of tourists, causing a negative impact on the image perception of ice and snow tourism destinations. If the service does not match the demand of tourists, the interest in IST brought by the Winter Olympics will also be offset. Therefore, for the sustainable development of IST, attention should be paid to the improvement of supporting levels of services, infrastructure, etc.

#### 5.2.2 Practical implication

*From a managerial perspective*. The development of ice and snow tourism destination is attributed to the feature of resources. Some spots with high slop, such as Park City Ski Resort in the United States, Verbier Ski Resort in Switzerland, Whistler Mountain Ski Resort in Canada, and Rosa Khutor Ski Resort in Russia, are suited for professional competition and training [84]. Others should pay more attention to the construction of snow field infrastructure, create tourism-supporting products, and provide consumers with a good skiing experience. Considering the high sensitivity of consumers to the price of snow equipment, a sharing economy mode of IST [85], such as Uber [86] and sharing umbrellas, can be put forward to promote the popularization of ice and snow sports. In addition, science and technology and electronic information technology can be used to make IST more intelligent and provide a better experience for tourists.

*From a social perspective*. Although IST is relatively mature in Europe, North America, and other regions and countries, for the emerging IST market like China, ice and snow activity is only popular in the limited region and ethnic minorities. In particular, the skiing has mostly not been attempted by residents in areas with underdeveloped ice resources, such as southern China. They felt strange and fear of ice and snow activities, and refuse to participate in [87]. Therefore, the government and other departments can improve tourist’s interest and happiness in participating in IST by building ice and snow places, holding ice and snow activities (festivals), media publicity, and ice and snow sports on campus, to achieve a balanced layout and sustainable development of IST.

### 5.3 Limitations and future research

There are some limitations in this study. Firstly, the research data excluded the evaluation from foreign tourists. Although the data was collected from popular Chinese websites such as Qunar, Ctrip, and Meituan with a large number of comments, due to language problems and usage habits, the experience perception evaluation of foreign tourists is missing. Secondly, the data type needs to be diversified. The information from photos [78], Weibo [88], and Twitter [89] can also help us insight the image perception of IST destinations. Thirdly, due to the influence of COVID-19 in early 2022, we cannot make field research, resulting in a lack of relevant questionnaires and interview data. In the future, we will visit relevant and typical destinations to deepen the results further. After all, this study examines the influence of the 2022 Winter Olympic Games on TDI in China. Despite there are large number of participants and a wide coverage of countries in this sporting event, it still cannot represent all the information in the world.

Future research should pay attention to the diversity of the data. The information from pictures and videos can enrich the text’s content and make the result more reliable. Moreover, the focus on the ecological impacts of IST sites development, such as animal habitats, environmental carrying capacity, etc., is important as well.

## Supporting information

S1 Data Data compression package. (RAR)

S1 File (ZIP)

S2 File (DOCX)
