# Supplementary material for: Image perception of ice and snow tourism in China and the impact of the Winter Olympics
Source: PLoS One. 2023 Jun 23;18(6):e0287530. doi: 10.1371/journal.pone.0287530 (PMC10289359; doi:10.1371/journal.pone.0287530)
Supplement: S2 File — (DOCX) [file pone.0287530.s003.docx]

**Supporting information**

**S1 table. Summary of Web text analysis results**

**Table 1 Comparative analysis of high-frequency words for Yabuli and Xiling**

| Yabuli New Sports Commission Ski Resort | | | | Xiling Snow Mountain | | | |
| --- | --- | --- | --- | --- | --- | --- | --- |
| Serial number | Glossary | Wordiness | Word Frequency | Serial number | Glossary | Wordiness | Word Frequency |
| 1 | Skiing | 1408 | verbs | 1 | Snowy Mountains | 825 | noun |
| 2 | Coaches | 1006 | noun | 2 | Ropeways | 824 | noun |
| 3 | Ski Resorts | 985 | noun | 3 | Scenery | 664 | noun |
| 4 | Yabuli | 785 | noun | 4 | Skiing | 520 | verbs |
| 5 | Services | 512 | verbs | 5 | Landscapes | 518 | noun |
| 6 | Fun | 421 | verbs | 6 | Scenic areas | 501 | noun |
| 7 | Experience | 366 | noun | 7 | Fun | 489 | verbs |
| 8 | Happy | 353 | verbs | 8 | Queuing | 401 | verbs |
| 9 | Harbin | 350 | noun | 9 | Sun and Moon | 389 | noun |
| 10 | Snow Trails | 347 | noun | 10 | Chengdu | 357 | noun |
| 11 | Hours | 346 | noun | 11 | Hours | 345 | noun |
| 12 | Tipping | 320 | noun | 12 | Weather | 336 | noun |
| 13 | Playful | 291 | verbs | 13 | Hilltop | 336 | noun |
| 14 | Scenery | 268 | noun | 14 | Projects | 325 | noun |
| 15 | Value for money | 223 | noun | 15 | Tickets | 319 | noun |
| 16 | Overall | 222 | noun | 16 | Gondola | 305 | noun |
| 17 | Time | 218 | noun | 17 | Experience | 303 | verbs |
| 18 | Interesting | 209 | adjectives | 18 | Worthwhile | 296 | verbs |
| 19 | Junior | 204 | distinguishing words | 19 | Time | 288 | noun |
| 20 | First time | 201 | numerals | 20 | On the Mountain | 283 | noun |
| 21 | Hot Springs | 196 | noun | 21 | Skiing | 274 | noun |
| 22 | Sports Commission | 184 | noun | 22 | Convenient | 269 | adjectives |
| 23 | Local | 179 | noun | 23 | Sea of Clouds | 267 | noun |
| 24 | Personnel | 174 | noun | 24 | Up the hill | 265 | verbs |
| 25 | Convenient | 174 | adjectives | 25 | Happy | 240 | verbs |
| 26 | Tourism | 168 | verbs | 26 | Beautiful | 237 | adjectives |
| 27 | Facilities | 160 | noun | 27 | Interesting | 237 | adjectives |
| 28 | Slipway | 159 | noun | 28 | Overall | 231 | noun |
| 29 | Recommendations | 159 | verbs | 29 | Local | 230 | noun |
| 30 | Attitude | 155 | noun | 30 | Recommendations | 221 | verbs |

**Table 2 Comparison of the emotional image before the Winter Olympics**

| Name | Positive sentiment | Neutral sentiment | Negative sentiment | Total number of statements |
| --- | --- | --- | --- | --- |
| Yabuli ice and snow tourism destination | 63.27% | 0.82% | 35.91% | 3907 |
| Chengdu Xiling ice and snow tourism destination | 61.91% | 0.97% | 37.12% | 5057 |

**Table 3 High-frequency words for Xiling before and after the Winter Olympics**

| List of high-frequency words for snow and ice tourism destinations for Xiling | | | | | | |
| --- | --- | --- | --- | --- | --- | --- |
| Serial number | Ahead of the 2022 Winter Olympics | | | After the 2022 Winter Olympic Games | | |
|  | Characteristic words | Proportion | Wordiness | Characteristic words | Proportion | Wordiness |
| 1 | Snowy Mountains | 0.1630 | noun | Snowy Mountains | 0.1621 | noun |
| 2 | Ropeways | 0.1629 | noun | Skiing | 0.1578 | verbs |
| 3 | Scenery | 0.1312 | noun | Queuing | 0.1536 | verbs |
| 4 | Skiing | 0.1028 | verbs | Scenic areas | 0.1284 | noun |
| 5 | Landscapes | 0.1024 | noun | Chengdu | 0.1052 | noun |
| 6 | Scenic areas | 0.0990 | noun | Ropeways | 0.0968 | noun |
| 7 | Fun | 0.0966 | adjectives | Landscapes | 0.0905 | noun |
| 8 | Queuing | 0.0792 | verbs | Hours | 0.0757 | noun |
| 9 | Sun and Moon | 0.0769 | noun | Downhill | 0.0631 | verbs |
| 10 | Chengdu | 0.0705 | noun | Skiing | 0.0055 | noun |
| 11 | Hours | 0.0682 | noun | Up the hill | 0.0589 | verbs |
| 12 | Weather | 0.0664 | noun | Experience | 0.0589 | noun |
| 13 | Hilltop | 0.0664 | noun | Time | 0.0547 | noun |
| 14 | Projects | 0.0642 | noun | Services | 0.0505 | verbs |
| 15 | Tickets | 0.0630 | noun | Beautiful | 0.0505 | adjectives |
| 16 | Gondola | 0.0603 | noun | Tickets | 0.0505 | noun |
| 17 | Experience | 0.0599 | verbs | Child | 0.0505 | noun |
| 18 | worthwhile | 0.0585 | verbs | Fun | 0.0484 | adjectives |
| 19 | Time | 0.0569 | noun | Convenient | 0.0484 | adjectives |
| 20 | On the Mountain | 0.0559 | noun | Gondola | 0.0484 | noun |
| 21 | Skiing | 0.0541 | noun | Projects | 0.0442 | noun |
| 22 | Convenient | 0.0531 | adjectives | Weather | 0.0442 | noun |
| 23 | Sea of Clouds | 0.0527 | noun | Facilities | 0.0442 | noun |
| 24 | Up the hill | 0.0523 | verbs | Hilltop | 0.0442 | noun |
| 25 | Happy | 0.0474 | adjectives | Sea of Clouds | 0.0421 | noun |
| 26 | Beautiful | 0.0468 | adjectives | Sun and Moon | 0.0421 | noun |
| 27 | Interesting | 0.0468 | Adjectives | Scenery | 0.0421 | noun |
| 28 | Overall | 0.0456 | noun | Go up | 0.0400 | verbs |
| 29 | Local | 0.0454 | noun | Snowscape | 0.0378 | noun |
| 30 | Recommendations | 0.0436 | verbs | Playful | 0.0357 | verbs |

Note: Frequency = word frequency / total number of words in the sample.

**Table 4 High-frequency words for Yabuli before and after the Winter Olympics**

| High-frequency word list for Yabuli IST tourism destination | | | | | | |
| --- | --- | --- | --- | --- | --- | --- |
| Serial number | Ahead of the 2022 Winter Olympic Games | | | After the 2022 Winter Olympic Games | | |
|  | Characteristic words | Proportion | Wordiness | Characteristic words | Proportion | Wordiness |
| 1 | Skiing | 0.3601 | verbs | Yabuli | 1.7272 | noun |
| 2 | Coaches | 0.2572 | noun | Skiing | 1.6262 | noun |
| 3 | Skiing | 0.2519 | noun | Skiing | 1.5757 | verbs |
| 4 | Yabuli | 0.2007 | noun | Harbin | 0.6868 | noun |
| 5 | Services | 0.1309 | verbs | Sports Commission | 0.5151 | noun |
| 6 | Fun | 0.1076 | verbs | Tourism | 0.4797 | noun |
| 7 | Experience | 0.0936 | noun | Resort area | 0.3989 | noun |
| 8 | Happy | 0.0902 | verbs | Snow Trails | 0.3787 | noun |
| 9 | Harbin | 0.0895 | noun | Snow and ice | 0.3232 | noun |
| 10 | Snow Trails | 0.0887 | noun | China | 0.3181 | noun |
| 11 | Hours | 0.0884 | noun | Sunshine | 0.3030 | noun |
| 12 | Tipping | 0.0818 | noun | Holiday Village | 0.2373 | noun |
| 13 | Playful | 0.0744 | verbs | Hotels | 0.2020 | noun |
| 14 | Scenery | 0.0685 | noun | Scenic areas | 0.1868 | noun |
| 15 | Value for money | 0.0570 | noun | Hilltop | 0.1818 | noun |
| 16 | Overall | 0.0567 | noun | Architecture | 0.1818 | noun |
| 17 | Time | 0.0557 | noun | Hours | 0.1767 | noun |
| 18 | Interesting | 0.0534 | adjectives | Coaches | 0.1616 | noun |
| 19 | Junior | 0.0521 | distinguishing words | Heilongjiang | 0.1616 | noun |
| 20 | First time | 0.0514 | numerals | Kilometers | 0.1565 | noun |
| 21 | Hot Springs | 0.0501 | noun | Experience | 0.1515 | noun |
| 22 | Sports Commission | 0.0470 | noun | Arrival | 0.1414 | noun |
| 23 | Local | 0.0457 | noun | Junior | 0.1414 | verbs |
| 24 | Personnel | 0.0445 | noun | Snow | 0.1414 | noun |
| 25 | Convenient | 0.0445 | adjectives | Web | 0.1313 | noun |
| 26 | Tourism | 0.0429 | verbs | North East | 0.1262 | noun |
| 27 | Facilities | 0.0409 | noun | Tickets | 0.1262 | noun |
| 28 | Slipway | 0.0406 | noun | Hot Springs | 0.1212 | noun |
| 29 | Recommendations | 0.0406 | verbs | Suitable for | 0.1212 | adjectives |
| 30 | Attitude | 0.0396 | noun | Winter | 0.1161 | noun |

Note(s): Frequency = word frequency / total number of words in the sample.

**Table 5 Comparison of the emotional image after the Winter Olympics**

| Name | Time | Positive sentiment | Neutral sentiment | Negative sentiment | Total number of statements |
| --- | --- | --- | --- | --- | --- |
| Yabuli ice and snow tourism Destination | Before the Winter Olympics | 63.27% | 0.82% | 35.91% | 3907 |
|  | After the Winter Olympics | 57.58% | 1.52% | 40.90% | 198 |
| Xiling ice and snow tourism Destination | Before the Winter Olympics | 61.91% | 0.97% | 37.12% | 5057 |
|  | After  the Winter Olympics | 49.26% | 1.26% | 49.48% | 475 |

**Table 6 Representative Positive and Negative Evaluation Texts**

|  | Positive Evaluation | Negative Evaluation |
| --- | --- | --- |
| Yabuli ice and snow tourism Destination | It’s worth going, the snow park lift facilities are good quality, the price is not too expensive, and the intermediate snow track is about 1 km long, ski down more comfortably. | Today’s theme is skiing at the new Sports Commission ski resort in Yabuli. They have hired a private instructor for each of the kids for 240 two hours each. The experience was bad. In the beginning, they did not let the two warm up and did not teach them to turn the brakes on the beginner track. As soon as they came up, they took the children directly to the gondola on the intermediate road and came down after a lap slide experience. The child said it was too cold to play. It was only half an hour before and after, and then they asked for tips, which disgusted me that the ski ticket was 280 for on-site purchase, 138 for Ctrip, and 120 for local people. |
|  | The weather in the Northeast is so cool, it's like a refrigerator. It feels super good to cross the New Year here, praying for 2021 to come true. Started my first ski trip to Yabuli in the New Year, very happy day, and all the services are very satisfactory. | This time to Yabuli skiing, with the group to go, travel agency pit, and a bunch of self-funded projects. What we went to was not the new sports committee written in the contract, but sunshine ski resort. The primary ski slopes open to tourists are not as big as Liaoyang Gongchangling ski resorts. The temperature here is shallow, hands cannot take out, and a group of Anhui friends is in the hall until the return journey. |
| Xiling ice and snow tourism Destination | It is really worth going to play ah, Sichuan locals and next door to Chongqing friends feel very nice! Sitting on the ropeway, the scenery is stunning, the world in front of you in silver, as if in a fairy tale world. The first section of the ropeway to the Yuanyang pool, more people, play a lot of places, and the visitor center in the snow is simply beautiful. You can also take a lot of good pictures! | Service is not good. No one answered the customer service number if you had a question. There was no one to keep order in the queue at the attraction. The environment is ok, but the service is terrible. |
|  | Xiling Snow Mountain is worth a visit! The snow is beautiful! It’s fun! Buy a set of tickets for parents and children! A family to play to buy a set of tickets is cost-effective, with children not tired. Scenic queuing orderly! Buy tickets online, do not have to queue on site, very time saving, to the site cell phone scan code to apply for the mountain queue number, or pay attention to the Xiling Snow Mountain public number in advance, and apply directly to the mountain queue number, very convenient. | One person just went less than a week. Just drop the price to 188, but also send two hours of skiing, the price difference is so big, garbage. The feeling of being cheated, we five people went to a loss of 600, garbage. On the ropeway, the scenery can still be. There is nothing else to play. Skiing is also dead expensive, and things are all rented separately. I have not seen the snow and can play under it, but be sure not to buy expensive ones! |

**S2.** **The reason why not use the LDA data analysis**

Chinese ice and snow tourism destinations have only received consumer attention in recent years, the content and number of related travelogues exist. However, given that Chinese ice and snow tourism destinations have only recently received consumer attention, there is a gap in the content and quantity of relevant travelogues, and many short textual travelogues will not apply to the LDA model approach.

**S3 dataset**

**1. Data crawling tool**

Octopus Collector Professional Edition 8.lnk.

**2. Date processing tool**

ROST Content Mining System: software for Content Mining and Analysis. Wuhan University, Hubei China.

**3. Website and URL used**

The data that support the findings of this study are available in [Meituan.com] at [https://nc.meituan.com/], in [Qunar.com] at [https://www.qunar.com/], in [Ctrip.com] at [https://www.ctrip.com/], in [Weibo.com] at [https://m.weibo.cn/], in [MaHoneycomb.com] at [https://www.mafengwo.cn/].

**4. Specific content of data collection**

| **Website** | **Keyword** | **Data Date** | **Data collection date** | **Set up** | **Data content** |
| --- | --- | --- | --- | --- | --- |
| https://nc.meituan.com/  https://www.qunar.com/  https://www.ctrip.com/  https://www.mafengwo.cn/  https://m.weibo.cn/ | Yabuli Ski Resort  Yabuli Qingyun Ski Resort  Yabuli Forest Park  Yabuli New Sports Committee  Yabuliyawance Ski Resort  Yabuli Sunshine Resort Ski Resort  Xiling Snow Mountain  Xiling Dafeishui Scenic Area  Xiling Ski Resort  Xiling Forest Park | From January 2018 to December 2021 | April 2022 | 1.Automatically skip invalid circular clicks  2.Browser version: Firefox web version 54.0  3.The cloud collection IP region is set as the default | 1.User name  2. Scoring  3.Comment time  4.Comments |
| https://nc.meituan.com/  https://www.qunar.com/ | Yabuli Qingyun Ski Resort  Xiling Snow Mountain | From February to April 2022 | May 2022 |  |  |

**5. Statement**

All data collection methods, including the use of potential crawlers, comply with the terms and conditions of the use source.
